# Effect of Selenium on Control of Postharvest Gray Mold of Tomato Fruit and the Possible Mechanisms Involved

**DOI:** 10.3389/fmicb.2015.01441

**Published:** 2016-01-06

**Authors:** Zhilin Wu, Xuebin Yin, Gary S. Bañuelos, Zhi-Qing Lin, Zhu Zhu, Ying Liu, Linxi Yuan, Miao Li

**Affiliations:** ^1^Key Laboratory of Agri-Food Safety of Anhui Province and Laboratory of Quality and Safty Risk Assessment for Agricultural Products on Storage and Preservation of the Ministry of Agriculture (Hefei), School of Plant Protection – School of Resources and Environment, Anhui Agricultural UniversityHefei, China; ^2^School of Earth and Space Sciences, University of Science and Technology of ChinaHefei, China; ^3^Jiangsu Bio-Engineering Research Centre of SeleniumSuzhou, China; ^4^Institute of Advanced Technology, University of Science and Technology of ChinaHefei, China; ^5^Water Management Research Unit, United States Department of Agriculture – Agricultural Research Service, ParlierCA, USA; ^6^Environmental Sciences Program and Department of Biological Sciences, Southern Illinois University Edwardsville, EdwardsvilleIL, USA; ^7^School of Chemistry and Biological Engineering, University of Technology and Science BeijingBeijing, China

**Keywords:** *Botrytis cinerea*, selenium, postharvest treatment, reactive oxygen species, membrane integrity, cellular leakage

## Abstract

Selenium (Se) has important benefits for crop growth and stress tolerance at low concentrations. However, there is very little information on antimicrobial effect of Se against the economically important fungus *Botrytis cinerea*. In the present study, using sodium selenite as Se source, we investigated the effect of Se salts on spore germination and mycelial growth of the fungal pathogen *in vitro* and gray mold control in harvested tomato fruit. Se treatment at 24 mg/L significantly inhibited spore germination of the fungal pathogen and effectively controlled gray mold in harvested tomato fruit. Se treatment at 24 mg/L seems to induce the generation of intracellular reactive oxygen species in the fungal spores. The membrane integrity damage was observed with fluorescence microscopy following staining with propidium iodide after treatment of the spores with Se. These results suggest that Se has the potential for controlling gray mold rot of tomato fruits and might be useful in integrated control against gray mold disease of postharvest fruits and vegetables caused by *B. cinerea*. The mechanisms by which Se decreased gray mold decay of tomato fruit may be directly related to the severe damage to the conidia plasma membrane and loss of cytoplasmic materials from the hyphae.

## Introduction

Mineral nutrients has important benefits for the growth and development of many organisms, and are essential factors in biotic plant interactions while influencing plant health (Ahn et al., 2005; Datnoff et al., 2007; Cabot et al., 2013; Crane et al., 2014). The most recent information regarding the effect of nutrients on disease resistance and tolerance to pathogens has been reported by others (Boyd, 2007; Fones et al., 2010; Hörger et al., 2013). Specific micronutrients (Cu, Fe, Mn, Zn, and B) reportedly affect many plant diseases (Dordas, 2008; Yao et al., 2012; Fones and Preston, 2013). In addition, some relatively non-toxic organic and inorganic salts such as potassium silicate and calcium chloride have been investigated for inhibition of fungal pathogens on fruits, vegetables, field crops, and ornamentals (Hervieux et al., 2002; Shi et al., 2011; Yaganza et al., 2014). Though selenium (Se) has been found to be beneficial to plants, its effect on plant–disease interaction has little been evaluated, and there are few investigations on Se salt treatment for control of plant diseases (Hanson et al., 2003; Trumble and Sorensen, 2008; Companioni et al., 2012).

Selenium is an essential trace element for humans and animals but at high concentrations, Se becomes toxic to organisms due to Se replacing sulfur in proteins (Zhao and McGrath, 2009; Zhu et al., 2009; Hasanuzzaman et al., 2010, 2014; Wu et al., 2015). The range between beneficial and harmful Se concentrations is relatively narrow for humans and animals. The minimal Se concentration in livestock feed is 0.05–0.10 mg/kg dry forage, while the toxic Se concentration in animal feed is 2–5 mg/kg dry forage. In humans, the World Health Organization (WHO) and USDA recommended the required human dietary intake of Se to be 55–200 μg/day for adults (Wu et al., 2015). Se is of great interest in many facets of biomedicine, biochemistry, and environmental science (Tapiero et al., 2003; Bañuelos, 2006; Doucha et al., 2009; Winkel et al., 2011; Yuan et al., 2013). Moreover, Se is used for obtaining more bioactivity and safety for chemical pesticide (Ma et al., 2006; Singh, 2012). Selenium sulfide and sodium selenite have been tested for inhibition of some pathogens (Brotherton, 1967; Wu et al., 2014). A mixture fungicide of Dithane and sodium selenite was more effective against *Aspergillus funiculosus* and *Alternaria tenuis* (Razak et al., 1991). The application of Se at low concentrations as possible alternatives to synthetic fungicides for the control of plant diseases, may reduce the potential hazardous effect on the environment and human health (Hasanuzzaman et al., 2010; Wu et al., 2014).

Gray mold decay, caused by species of *Botrytis cinerea*, is one of the most important postharvest diseases of fresh fruit and vegetables worldwide (Qin et al., 2010; Youssef and Roberto, 2014). Infections by *B. cinerea* that could lead to severe economic losses both at pre- and post-harvest stages (Soylu et al., 2010; Cabot et al., 2013). Currently, the use of synthetic chemical fungicides is the primary means to control postharvest diseases (Zhu et al., 2010). However, growing concern for the potential impact of fungicide residues on the environment and human health risks, the development of fungicide-resistant strains of pathogen, and the lack of the most effective fungicides have created an interest in exploring for alternative approaches for the disease management (Janisiewicz and Korsten, 2002). Among alternative control methods, the application of antagonistic microorganisms, and the use of bicarbonates, chitosan, hot water, essential oils, either alone or in combination with other treatments as part of the integrated management of postharvest diseases strategies, have been investigated for the control of postharvest decay of fruits and vegetables with some success (Karabulut et al., 2004, 2005; Gabler et al., 2005; Droby et al., 2009; Soylu et al., 2010; Zhang et al., 2014; de Souza et al., 2015). However, there is a need for developing alternative methods to control postharvest gray mold of fruits and vegetables that are safe, effective, economical, and compatible with value-added practices such as producing Se-biofortified fruits and vegetables.

Our previous studies showed that sodium selenite could control postharvest disease of fruits and vegetables caused by *Penicillium expansum* (Wu et al., 2014). However, to our knowledge, little information is available concerning the antimicrobial activity of sodium selenite against postharvest pathogen *B. cinerea* and that the mechanism of action still remains essentially unexplored. Therefore, we aimed to explore the potential of using sodium selenite as an antifungal agent for the control of *B. cinerea* that causes gray mold disease on postharvest fruits and vegetables. In the present study, the experiments *in vitro* and *in vivo* were designed with sodium selenite treatments to directly inhibit *B. cinerea*, and to investigate the control of gray mold disease on postharvest tomato fruit. The underlying mechanisms of action by which sodium selenite caused fungal death, and affected intracellular reactive oxygen species (ROS) and the integrity of the plasma membrane of *B. cinerea* were also assessed.

## Materials and Methods

### Pathogen and Chemicals

The postharvest fungal pathogen *B. cinerea* used in this study was obtained from Key Laboratory of Plant Resources, Institute of Botany, Chinese Academy of Sciences. The fungus was inoculated and re-isolated from tomato fruit to maintain pathogenicity. The isolates were routinely grown on potato dextrose agar (PDA) plates for 2 weeks at 23°C in the darkness. The spores were obtained from the surface of the agar and suspended in 5 mL of sterile distilled water containing 0.1% v/v Tween 20. Spore suspensions were filtered through four layers of sterile cheesecloth to remove mycelia fragments. A hemocytometer was used to calculate the number of spores and the spore concentration was adjusted to 5.0 × 10^5^ spores/mL before conducting subsequent experiments.

Sodium selenite used as Se source in this study and was purchased from Sigma–Aldrich (St. Louis, MO, USA). Propidium Iodide (PI), 2,7-dichlorodihydrofluorescein diacetate (DCHF-DA) were purchased from Sigma-Aldrich (St. Louis, MO, USA). All other chemicals used in this study were of high analytical grade.

### Inhibitory Effect of Se on Spore Germination and Germ Tube Elongation of *B. cinerea*

The effect of sodium selenite on spore germination and germ tube elongation of *B. cinerea* was assayed in potato dextrose broth (PDB) medium following the method described by Qin et al. (2010). In brief, aliquots of a spore suspension of *B. cinerea* were added to wells of a 24-well microtitration plate containing PDB medium to obtain a final concentration of 5 × 10^5^ spores/ml. The culture medium was supplemented with different concentrations of sodium selenite at 0, 6, 12, 18, 24 mg Se/L at pH of 7.0, which are referred to Razak et al. (1991) and Wu et al. (2014, 2015). The microtitration plate was incubated at 23°C on a rotary shaker at 200 rpm. About 200 spores were examined microscopically for germination level and germ tube length after 8 h of incubation. Spores were considered germinated if the germ tube was equal to or greater than the diameter of the spore. Germ tube length was determined with an ocular micrometer and germinated spores were expressed as percentage of the total number of evaluated spores. There were three replicates for each treatment, and the experiment was repeated twice.

### Effect of Se on Mycelial Growth of *B. cinerea*

The effect of sodium selenite on mycelial growth of *B. cinerea* was assayed in PDA following the method described by Qin et al. (2010). A 5-mm diameter plug of mycelial agar was obtained from the growing edge of 7-day-old cultures of the fungi and placed in the center of a 9-cm-diameter petri dish containing PDA medium with different concentrations (0, 6, 12, 18, 24 mg Se/L) as sodium selenite solutions. Sodium selenite solutions were filtered through a 0.45 μm Millipore filter before they were, respectively, added to the autoclaved PDA medium that had cooled to 60°C. Results are reported as the diameter of the fungal colony (mycelium) in the petri dish minus the diameter of the agar plugs (5 mm). Radial growth of *B. cinerea* was observed after incubation at 23°C for 3, 4, 5, 6, and 7 days, respectively. Radial growth of *B. cinerea* was calculated as the average of the orthogonal diameter, the results were expressed as minimum inhibitory concentration (MIC) according to Arslan et al. (2009). Each treatment contained three replicates and the entire experiment was repeated twice.

### Reactive Oxygen Species (ROS) Assay

The oxidant-sensitive probe DCHF-DA was used to assess the intracellular ROS levels in *B. cinerea* according to Shi et al. (2012) with some modifications^.^ Spores of *B. cinerea* were cultured in PDB medium supplemented with 0 or 24 mg/L sodium selenite and incubated for 1 and 3 h. The spores were washed with 10 mM potassium phosphate buffer (pH 7.0) and incubated for 1 h in the same buffer containing 10 μM DCHF-DA (dissolved in dimethyl sulfoxide). After washing twice with potassium phosphate buffer, spores were examined under a AMG microscope (AMG, EVOS-S1, USA) using a fluorescein 2,7-dichlorodihydro-specific filter. At least 100 spores were examined for each treatment with three replications.

### Membrane Integrity Assay and Microscopy

The fluorescence microscopy following staining with PI was used to assess the membrane integrity of *B. cinerea* conidia according to the methods of Wu et al. (2014) with some modifications. Spores of *B. cinerea* were cultured in PDB medium supplemented with 0 and 24 mg/L sodium selenite and incubated for 1, 3, and 5 h. Then, the spores were collected and stained with 10 μg/ml PI for 5 min at 30°C. Because PI is membrane impermeant, only cells that have lost membrane integrity will show red staining. The spores were centrifuged and washed twice with 10 mM potassium phosphate buffer (pH 7.0) to remove residual dye. The spores were then observed on a AMG microscope (AMG, EVOS-S1, USA) equipped with individual fluorescein rhodamine filter set (excitation BP 546/12 nm, emission LP 590 nm), and the images were collected. At least 100 spores were examined for each treatment with three replications.

### Cellular Leakage Assay

The leakage of cytoplasmic contents from mycelium of *B. cinerea* was determined according to the methods of Lai et al. (2011) with some modifications. *B. cinerea* was grown in conical flasks (250 mL) containing 100 mL PDB medium at 23°C on a rotary shaker at 200 rpm and mycelia harvested after 3 days of incubation. After pooled and washed with sterile distilled water, the mycelia were re-suspended in 100 mL sterile distilled water containing 0–24 mg Se/L as sodium selenite, and incubated on a rotary shaker at 23°C for 1, 2, 3, and 4 h. Mycelia were then filtered from the solutions through a 0.2 μm pore size membrane and the aqueous solutions were used for determination of soluble proteins and carbohydrates. The Bradford assay was performed on the filtrates to quantify proteins by the various treatments (Bradford, 1976). Soluble carbohydrate was determined with anthrone reagent using glucose as the standard (Xu et al., 2006).

### Measurement of Cellular Enzymatic Activity

*Botrytis cinerea* was grown in conical flasks (50 mL) containing 20 mL PDB medium with 0–24 mg Se/L as sodium selenite at 23°C on a rotary shaker at 200 rpm and mycelia were harvested after 1, 2, and 3 days of incubation. The leakage of cytoplasmic contents from mycelium of *B. cinerea* was determined according to the method of Lai et al. (2011) with some modifications. The 0.5 g mycelia were filtered from the solutions and ground into homogenate with phosphate buffer (pH 6.8) and a small quantity of quartz sand in liquid nitrogen. The homogenate was centrifuged at 12000 rpm for 20 min and the supernatant was used for determination of enzymatic activity. The Bradford assay was performed on the supernatant to quantify release of proteins by the various treatments according to the method of Bradford (1976) with some modifications. The content of glutathione (GSH), methane dicarboxylic aldehyde (MDA), and superoxide dismutase (SOD) was determined with the previously reported method (Liu et al., 2005). Hydrogen peroxide (H_2_O_2_) and superoxide anion (O_2_^-^) production rate were determined with the methods of Xu et al. (2006).

### Effect of Se on Gray Mold Disease on Tomato Fruit

Seeds of tomato (*Lycopersicon esculentum* Mill. cv. Zhenbao) were obtained from the Academy of Agricultural Science in Anhui, China. During all experiments, tomato plants were greenhouse grown at Suzhou Key Advanced Laboratory for Selenium and Human Health, Suzhou Institute for Advanced Study, University of Science and Technology of China (Suzhou, China) with a mean temperature cycle of 31°C day/24°C night, relative humidity of 43%/85%, and a light period of 16 h, at 450–500 μmol/m^2^/s maximum photon flux densities that were measured at plant canopy level (IRGA, model LCA-4, Hoddesdon, UK). The fruits were harvested at commercial maturity (surface color of the fruit was red), and sorted based on size and the absence of physical injuries or disease infection. Before treatments, fruits were surfaced-disinfected with 2% sodium hypochlorite for 3 min, then rinsed with sterile water, and air-dried. The surface of the fruit was prepared for inoculation by inflicting a single 1-mm deep wound in the middle of each fruit with a sterile needle. Each wound was then inoculated with the pathogen *B. cinerea* by placing 10 μL of spore suspension (1 × 10^4^ spores/L) on the wound. The inoculated fruit were incubated overnight in a sterile box at 23°C before dipping it in the sodium selenite solution at concentration of 24 mg/L for 30 min. Fruit dipped in sterile distilled water served as the control. The treated fruit were incubated in a moist plastic box at 23°C for 6 days and disease development was assessed by measuring the lesion diameter and decay incidence of the gray mold on tomato fruit. Each treatment consisted of 50 fruits with three replications and the experiment was repeated twice.

### Statistical Analysis

All statistical analyses were performed using the SPSS software version 13.0 (SPSS Inc., Chicago, IL, USA) and analyzed by one-way analysis of variance (ANOVA). Mean separations were performed by Duncan’s multiple range tests. Differences at *p* < 0.05 were considered significant.

## Results

### Effects of Se on Spore Germination and Germ Tube Elongation of *B. cinerea*

As shown in **Table 1**, selenite was effective in inhibiting spore germination and germ tube elongation of *B. cinerea* following a concentration-dependent trend (*p* < 0.05). After incubation for 8 h at 23°C, germination of *B. cinerea* spores was strongly inhibited by selenite at the concentration of 24 mg/L.

**Table 1:** Effects of Se on spore germination and germ tube elongation of *Botrytis cinerea* after 8 h incubation at 23°C.

	CK (PDB)	Selenite (mg/L)			
		
		6	12	18	24
Spore germination (%)	96.4 ± 7.2^a^	79.1 ± 6.3^b^	43.6 ± 6.8^c^	12.3 ± 4.6^d^	4.9 ± 1.8^e^
Germ tube length (μm)	287.6 ± 46.4^a^	262.5 ± 34.6^a^	164.3 ± 15.8^b^	52.1 ± 6.2^c^	24.8 ± 5.1^d^


*Mean values followed by the different letters differ significantly within a row according to Duncan’s multiple range tests (*p* < 0.05), respectively.*

### Inhibitory Effect of Se on Mycelial Growth of *B. cinerea*

As shown in **Table 2**, sodium selenite was effective in inhibiting the mycelial growth of *B. cinerea* on PDA. After incubation for 3–7 days at 23°C, the growth of *B. cinerea* was significantly inhibited when the treatment concentration of sodium selenite was 24 mg/L (**Table 2**). Moreover, there was a significantly positive correlation between sodium selenite concentration and the effect on mycelial growth (**Table 2**).

**Table 2:** Radial growth inhibition of *Botrytis cinerea* by Se salts *in vitro*.

Selenite (mg/L)	Diameter of mycelial growth of *B. cinerea* (mm/days)				
	
	3 days	4 days	5 days	6 days	7 days
0 (CK)	37.8 , 6.3ˆa	61.3 , 11.2ˆa	84.6 , 5.3ˆa	90.0 , 0.3ˆa	90.0 , 0.1ˆa
6	25.2 , 5.1ˆb	41.5 , 8.6ˆb	50.8 , 6.5ˆb	68.4 , 6.3ˆb	86.6 , 5.1ˆa
12	10.4 , 4.8ˆc	20.1 , 4.8ˆc	26.4 , 4.7ˆc	35.7 , 4.2ˆc	50.3 , 6.0ˆb
18	5.6 , 1.4ˆd	8.3 , 3.1ˆd	13.8 , 3.6ˆd	21.6 , 3.3ˆd	32.9 , 4.3ˆc
24	5.1 , 0.8ˆd	6.4 , 1.9ˆd	8.2 , 2.7ˆe	11.1 , 2.1ˆe	15.5 , 3.0ˆd


*The diameter of *B. cinerea* was determined after 3–7 days incubation at 23 ° C on PDA amended with different selenite concentrations. Mean values followed by the different letters differ significantly within a row according to Duncan’s multiple range tests (*p* < 0.05), respectively.*

### Generation of Reactive Oxygen Species

**FIGURE 1:**
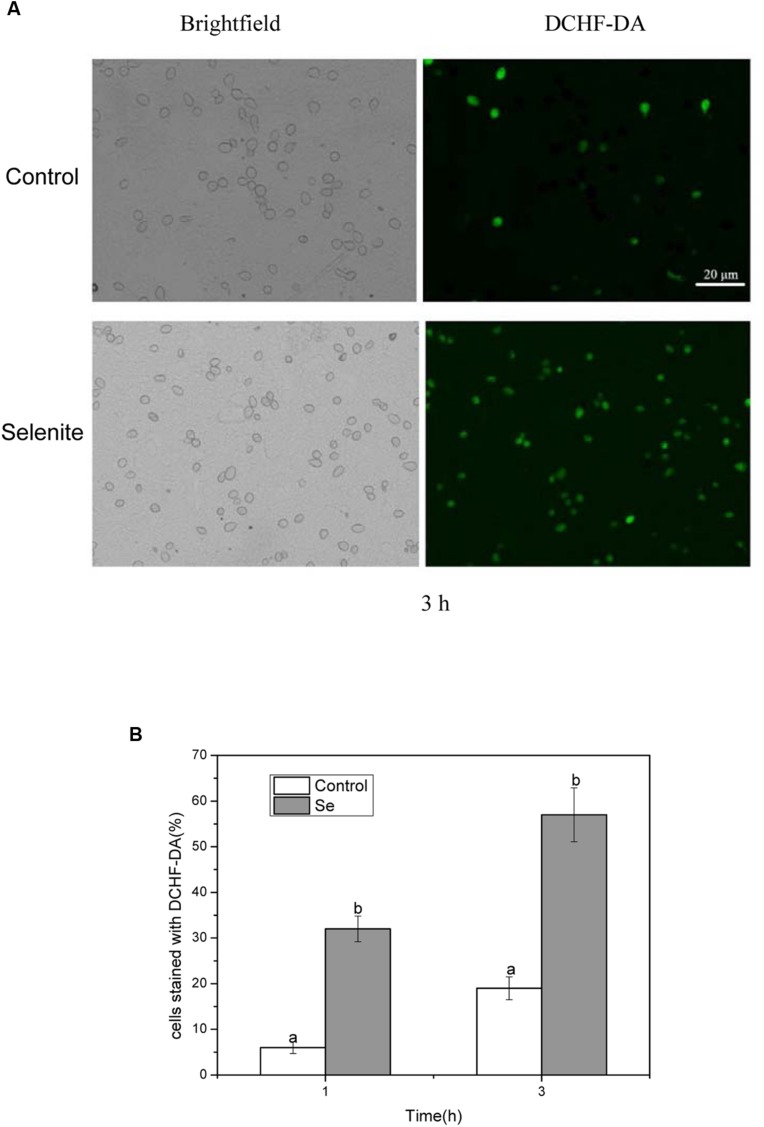
**(A)** Effect of selenite at 24 mg/L on production of reactive oxygen species in spores of *Botrytis cinerea*. **(B)** The percentage of spores stained with DCHF-DA. Spores incubated for 1 and 3 h. Vertical bars indicate standard deviations. Columns followed by different letters are statistically different according to the Duncan’s multiple range test (*p* < 0.05).

Reactive oxygen species generation was monitored when *B. cinerea* was exposed to 24 mg/L selenite for 1, 2, and 3 h at 23°C (**Figures 1A,B**). After 1 h of incubation, a majority of spores in the control treatment were not stained by DCHF-DA, implying poor ROS production at the time. However, in the selenite treatment about 32% of spores were stained by DCHF-DA (**Figure 1B**). With an increased incubation period, even higher levels of ROS were detected in Se-treated *B. cinerea* spores compared with the control. The highest percentage of stained spores (57%) was observed following 3 h of incubation with 24 mg Se/L, indicating that an increasing amount of oxidizing molecules was produced in cells exposed to selenite (**Figures 1A,B**).

### Assessment of Plasma Membrane Integrity by PI Staining

**FIGURE 2:**
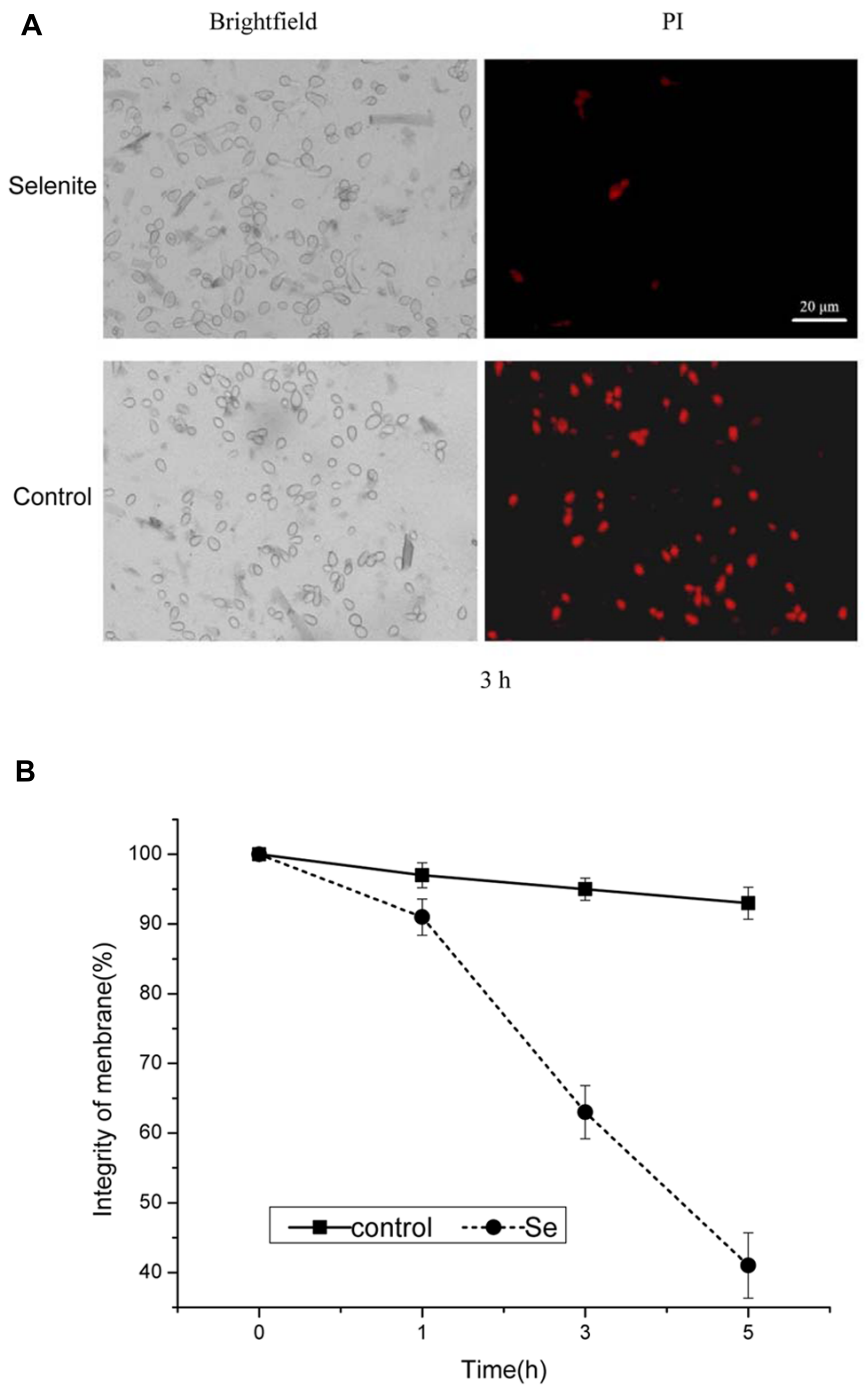
**(A)** Effect of selenite at 24 mg/L on plasma membrane integrity of *B. cinerea* conidia. **(B)** Percentage of plasma membrane integrity of *B. cinerea* spores. Spores incubated for 1, 3, and 5 h. Vertical bars indicate standard deviations. Columns followed by different letters are statistically different according to the Duncan’s multiple range test (*p* < 0.05).

The plasma membranes integrity was assessed by PI staining when *B. cinerea* was exposed to 24 mg/L selenite for 1, 3, and 5 h at 23°C (**Figures 2A,B**). After 1 h of incubation, the plasma membranes of *B. cinerea* were damaged under selenite stress (*p* < 0.05; **Figures 2A,B**). Membrane integrity of *B. cinerea* spores markedly declined with increasing incubation period in PDB containing selenite compared with the control (**Figures 2A,B**). Most (≥90%) untreated spores appeared to have intact plasma membranes, whereas the membrane integrity of spores decreased from 63% to 42% following 3 to 5 h of incubation with 24 mg Se/L (**Figures 2A,B**).

### Evaluation of Cellular Leakage

**FIGURE 3:**
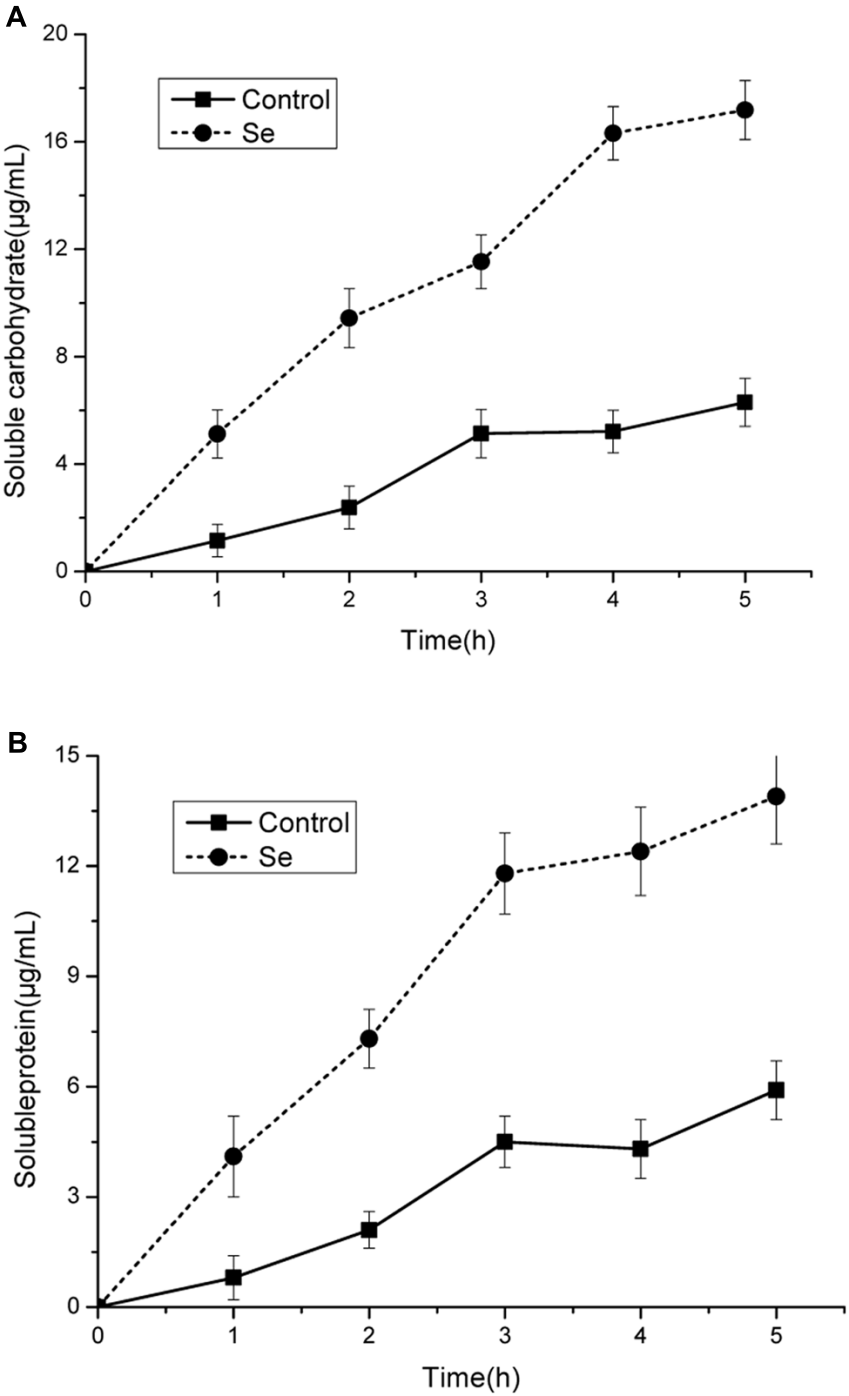
**Effect of selenite at 24 mg/L on leakage of carbohydrates **(A)** and protein **(B)** of *B. cinerea* mycelia cultured in sterile double-distilled water at 23°C.** Values for both soluble proteins and carbohydrates were expressed as μg/mL of aqueous solutions. Vertical bars indicate standard deviations.

The leakage of proteins and carbohydrates were significantly increased under selenite at 24 mg/L stress compared with the control (**Figures 3A,B**). These results suggests that loss of membrane integrity would lead to the release of cytoplasmic contents from the cells. The production of soluble protein and soluble carbohydrates were increased significantly when the damage of cell membrane integrity caused by the leakage of cellular constituents.

### Changes of SOD Activities, H_2_O_2,_ MDA, GSH content, and O_2_^-^ Production Rate

**FIGURE 4:**
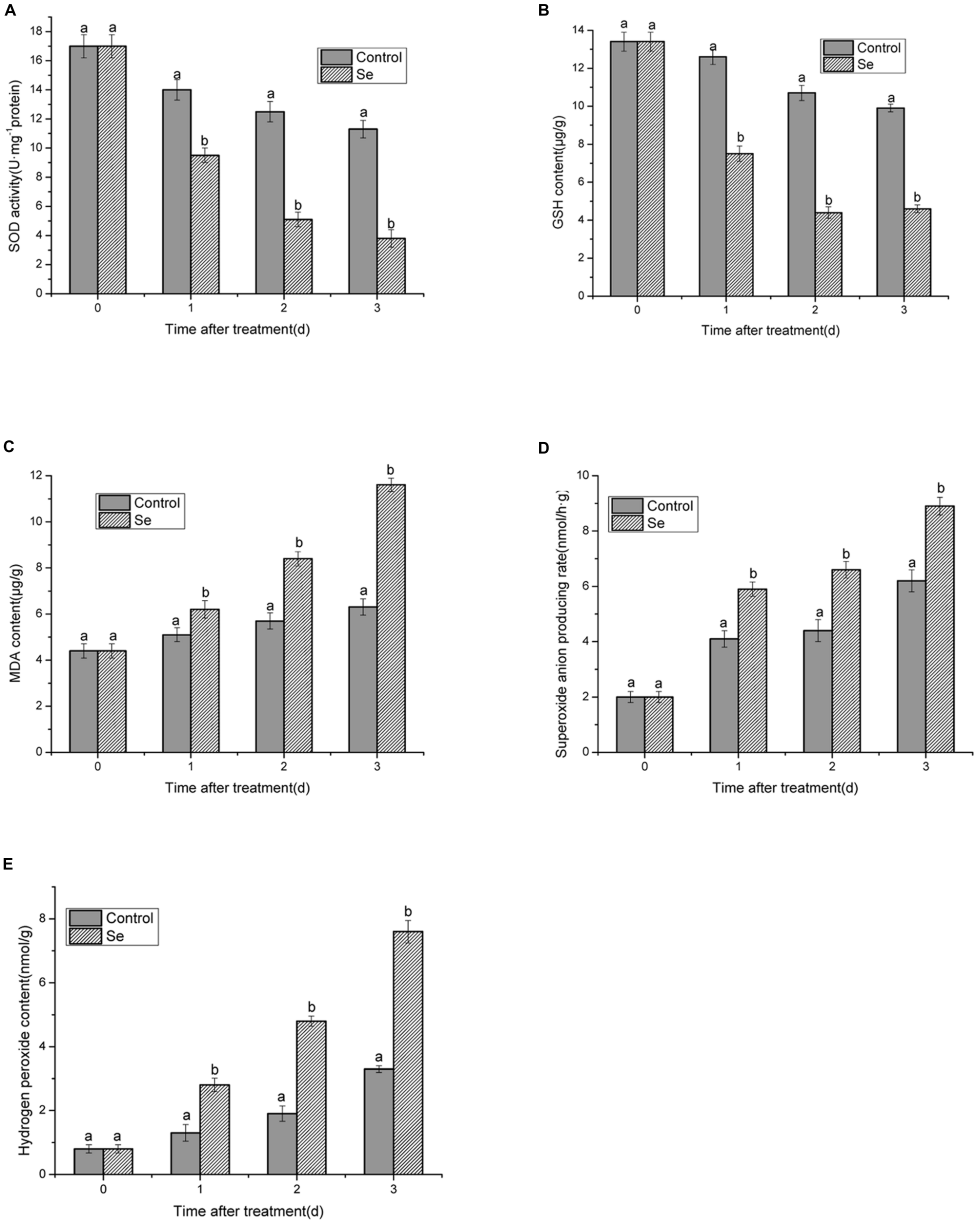
**Effects of selenite at 24 mg/L on activities of SOD **(A)**, GSH, and MDA content **(B,C)**, O_2_^-^ and H_2_O_2_ production rate **(D,E)** of *B. cinerea* spores after 1, 2, and 3 days of incubation at 23°C.** Vertical bars indicate standard deviations. Columns followed by different letters are statistically different according to the Duncan’s multiple range test (*p* < 0.05).

As shown in **Figures 4A,B**, both SOD activities and GSH content presented the similar decreasing trend from 1 to 3 days under selenite at 24 mg/L stress compared with the control. Moreover, upon exposure to selenite at 24 mg/L, the values of SOD and GSH were significantly lower than those of the control at each time point, and the decrease of SOD activity and GSH content was more fast than the control. Minimum values for SOD activity were 3.8 U/mg protein and 4.6 μg/g GSH content.

The MDA content significantly increased under selenite at 24 mg/L stress compared with the control, especially after 3 days with a maximum of 11.6 μg/g (**Figure 4C**). H_2_O_2_ content, and O_2_^-^ production rate of the control still increased, however, the production rate of them was clearly lower than that observed with selenite at 24 mg/L treatment. Rates increased to 8.9 nmol/g and 7.6 nmol/g for H_2_O_2_ and O_2_^-^, respectively (**Figures 4D,E**).

### Effect of Se on Gray Mold Disease on Tomato Fruit

**FIGURE 5:**
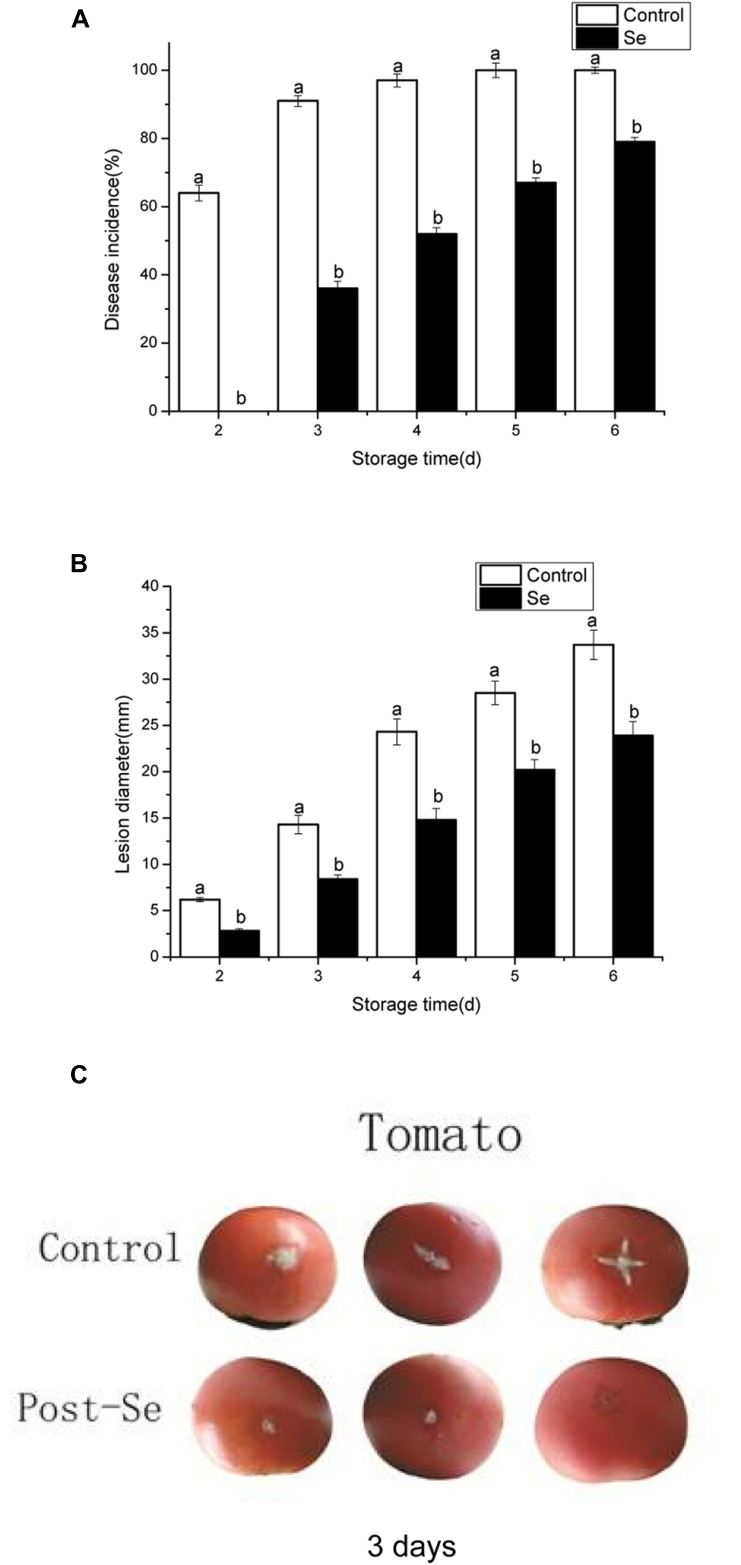
**Effect of selenite at 24 mg/L on disease incidence **(A)** and lesion diameter **(B)** of gray mold on tomato fruit cause by *B. cinerea* after 1–6 days incubation at 23°C.** Symptoms in inoculated tomato fruit stored in 23°C for 3 days **(C)**. Vertical bars indicate standard deviations. Mean values present in each bar followed by different letters are statistically different according to the Duncan’s multiple range test (*p* < 0.05).

Selenite at 24 mg/L was effective in inhibiting the lesion diameter of postharvest gray mold of tomato fruit for 6 days storage at 23°C. (*p* < 0.05). After inoculation of tomato fruit for 2 days, the control fruits started to decay, while the Se-treatment group did not show any disease symptoms. Compared with 100% disease incidence in the control, the disease incidence in the Se-treatment fruits was less than 80% (**Figure 5A**). After inoculation for 3 and 6 days, the lesion diameters were only 8 and 24 mm in the Se-treatment group, while the control group reached 14 and 34 mm, respectively (**Figures 5B,C**). These results indicated that sodium selenite was effective for postharvest gray mold control in tomato fruit (at the concentration of 24 mg Se/L).

### Comparison of the Inhibition Effects of Se with Several Inorganic Salts on *B. cinerea*

As shown in **Table 3**, Se could inhibit *B. cinerea* at minimum concentrations (e.g., Se at 0.012 g/L), as reported several inorganic salts such as silicate and borate, some of which have antimicrobial properties and could be useful as postharvest or preharvest treatments for gray mold disease control in the literature (Franck et al., 2005; Huang et al., 2008; Qin et al., 2010; Cabot et al., 2013; Harren and Tudzynski, 2013). This finding suggests that sodium selenite might serve as a potential alternative to synthetic fungicides for the reduction of the postharvest disease of fruit and vegetables caused by *B. cinerea*.

**Table 3:** Comparison of inhibitory effects of several similar inorganic salts on *Botrytis cinerea.*

Inorganic salts	Minimum inhibitory concentration(g/L)	Inhibitory fungal pathogen	Reference
Selenium	0.012	*Botrytis cinerea*	Current study
Boron	1.0	*Botrytis cinerea*	Qin et al., 2010
Sulfur	1.22	*Botrytis cinerea*	Franck et al., 2005
Silicon	0.042	*Botrytis cinerea*	Cabot et al., 2013
Calcium	2.0	*Botrytis cinerea*	Harren and Tudzynski, 2013


*Radial growth of *B. cinerea* was calculated as the average of the orthogonal diameter, the data were expressed as minimum inhibitory concentration (MIC) according to Arslan et al. (2009).*

## Discussion

Mineral nutrients increase plant growth and development while influencing plant health. Specific elements such as Si and B have reportedly affected many plant diseases (Fauteux et al., 2005; Datnoff et al., 2007; Qin et al., 2010). Moreover, some inorganic and organic salts could be useful as postharvest or/and pre-harvest treatments for postharvest diseases control (Hervieux et al., 2002; Shi et al., 2011; Yaganza et al., 2014). Among of these salts, silicon (Si), and Se are widely studied trace elements and have been found to be protective for plants under abiotic and biotic stress conditions (Fauteux et al., 2005; Ma and Yamaji, 2006; Liang et al., 2007; Hasanuzzaman et al., 2014; Rahman et al., 2015). Both Si and Se were reported to play roles in conferring oxidative stress tolerance by enhancement of the antioxidant defense system in plants. Si is the second most abundant element and its presence in the form of silicic acid allows its uptake by plants, however, those plants not supplied with sufficient natural sources of Si may benefit from its exogenous application (Fauteux et al., 2005; Ma and Yamaji, 2006; Liang et al., 2007; Hasanuzzaman et al., 2014; Rahman et al., 2015). Se, an essential element for animals and humans, has also been found to be beneficial to plants, which also plays a protective role in conferring tolerance to certain abiotic and biotic stresses when applied at lower concentrations, while higher concentrations show phytotoxicity (Zhao and McGrath, 2009; Zhu et al., 2009; Hasanuzzaman et al., 2010; Wu et al., 2014).

Recently, some studies demonstrate that Se has important physiological functions for plant growth and quality improvement in fruit at low concentrations (Zhao and McGrath, 2009; Zhu et al., 2009; Hasanuzzaman et al., 2010; Wu et al., 2015). In recent years, based on the elemental defense hypothesis in relation to pathogens, Se has been shown to exert a positive effect on stress tolerance and used to control plant diseases in some crops (Hanson et al., 2003; Quinn et al., 2010; Companioni et al., 2012; Hladun et al., 2013). Hanson et al. (2003) showed Se accumulation could protect plants against invertebrate herbivory damage and fungal infection. Se containing substances in leaves and stems of alfalfa and cruciferous crops act as repellents, so that aphids, caterpillars, and spider mites avoid these plants (Galeas et al., 2008; Quinn et al., 2010). Even in small amounts these substances are also toxic for these pests (Freeman et al., 2007, 2009, 2012; Galeas et al., 2008; Hladun et al., 2013). Moreover, Leaves of plant supplied with Se were less susceptible to both *Alternaria brassicicola* and a *Fusarium* sp. (Hanson et al., 2003). From experiments with Indian mustard (*Brassica juncea*) it appears that application of Se can enhance resistance to fungal diseases (Hanson et al., 2003). Se is not commonly added to nutrient solutions in tomato, but in a Mexican study (Companioni et al., 2012) application of sodium selenite reduced damage inflicted by *Fusarium wilt*. Additionally, total protein content and antioxidant activity increased in both susceptible and resistant cultivars of tomato.

In previous studies, we found that germination and germ tube elongation of *P. expansum* were significantly inhibited by the addition of 20 mg/L selenite to the PDB. In addition, we found that the mechanisms involved damage of Se to cellular oxygen-eliminating system (Wu et al., 2014). In the current study, we found that selenite significantly inhibited spore germination and germ tube elongation of *B. cinerea* (**Table 1**) and reduced the lesion diameter of gray mold disease in harvested tomato fruit after artificially inoculating pathogen spores (**Figure 5**). From these results we could conclude that selenite has a potential as a promising antifungal agent and also as a potential alternatives to synthetic fungicides in the protection of tomato fruit against *B. cinerea*, and selenite has a broadly inhibiting effect on postharvest fungal pathogens. The mechanism of action appeared to be closely related to the generation of active oxygen. ROS may cause oxidative damage to cellular compounds and lead to cellular dysfunction or cell death (Shi et al., 2012). In this study, we used an oxidant-sensitive probe DCHF-DA to investigate the ROS accumulation in *B. cinerea* spores, and observed that Se-treated spores showed a higher ratio of stained cells than that in control (**Figures 1** and **2**), indicating that Se induced ROS accumulation in *B. cinerea* spores. ROS accumulation can cause oxidative damage of the fungal spore and result in lower spore germination.

In addition, understanding the mode of action by which sodium selenite inhibits *B. cinerea* will help in the application of sodium selenite for management of pre- or/and postharvest gray mold disease of fruits and vegetables. Therefore, further studies are needed on the molecular mechanism of sodium selenite against *B. cinerea* by the “Omics approaches” (e.g., proteomics, metabolomics, genomics, transcriptomics), the effect of Se on nutrition and quality of tomato fruit, using confocal microscopy and TEM to confirm if selenite treatment could lead to mitochondrial damage, and the development of unique valued-added Se-enriched nutraceutical agricultural products from Se-biofortified fruits and vegetables, etc. Specifically, only sodium selenite and *B. cinerea* was tested in the study. Much more plant pathogenic fungal and bacterial species need to be tested to investigate if the antimicrobial effect of Se compounds such as sodium selenite and/or sodium selenate at low concentration against fungal and/or bacterial species is specific. Moreover, Se is one of essential micronutrients for human and animals health but at high concentrations, Se becomes toxic to vertebrates, especially birds, such that widespread use of this antimicrobial agent needs to be evaluated in the context of potential ecological impacts.

## Conclusion

In this study, sodium selenite at a concentration of 24 mg Se/L was effective in controlling gray mold in tomato fruits caused by *B. cinerea*. Moreover, we conclude that selenite treatment can stimulate ROS accumulation in fungal spores, resulting in oxidative damage, which may act as the antifungal mechanism of selenite inhibiting spore germination of *B. cinerea* and controlling gray mold in tomato fruits. In summary, we have shown that selenite could directly inhibit the growth of *B. cinerea in vitro*. Moreover, Se resulted in oxidative damage, which then played an important role in the inhibitory effect on *B. cinerea*. Additionally, application of selenite is promising as an alternative to synthetic fungicides for postharvest gray mold disease control in tomato fruit.

## Author Contributions

ZW and ML wrote the main manuscript text, and ZW and LY prepared all figures and tables. GB and Z-qL revised the manuscript. XY, YL, and ZZ have provided input and assistance to the submission of the final manuscript. All authors reviewed the manuscript.

## Conflict of Interest Statement

The authors declare that the research was conducted in the absence of any commercial or financial relationships that could be construed as a potential conflict of interest.
